# Supplementary material for: A high-throughput Galectin-9 imaging assay for quantifying nanoparticle uptake, endosomal escape and functional RNA delivery
Source: Commun Biol. 2021 Feb 16;4:211. doi: 10.1038/s42003-021-01728-8 (PMC7887203; doi:10.1038/s42003-021-01728-8)
Supplement: Supplementary file 2 — Supplemental Information [file 42003_2021_1728_MOESM2_ESM.pdf]

Supplementary Figure 1

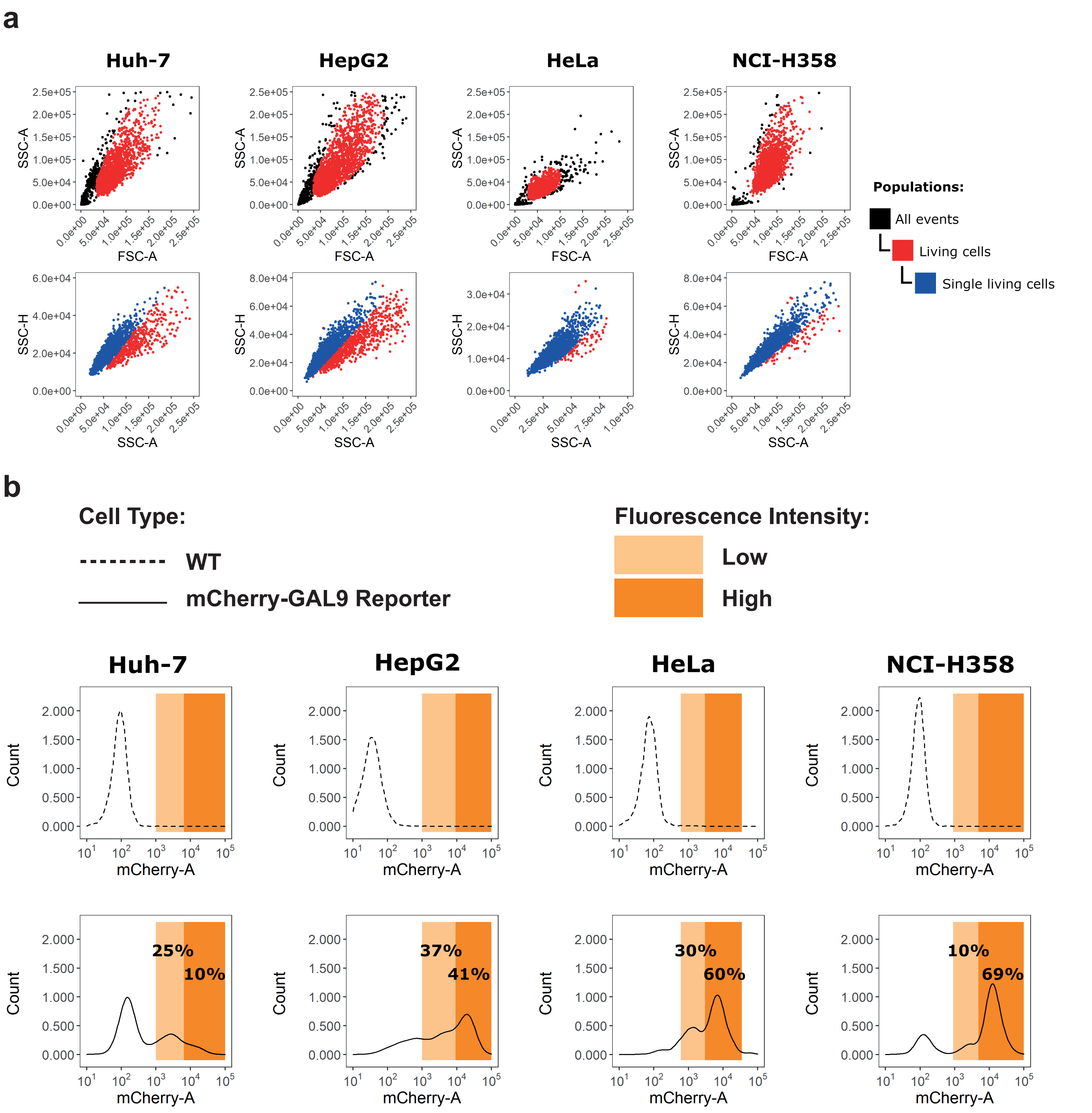

Supplementary Fig. 1 - Fluorescence-activated cell sorting

a Debris were excluded by plotting the FSC vs SSC with all events and gating for living cells, and cell aggregates (doublets/clusters) were excluded by plotting the SSC-height vs SSC-area with living cells and gating for single cells **b** mCherry expressing reporter cells were sorted to gate and select for highest cell reporter levels.

Supplementary Figure 2

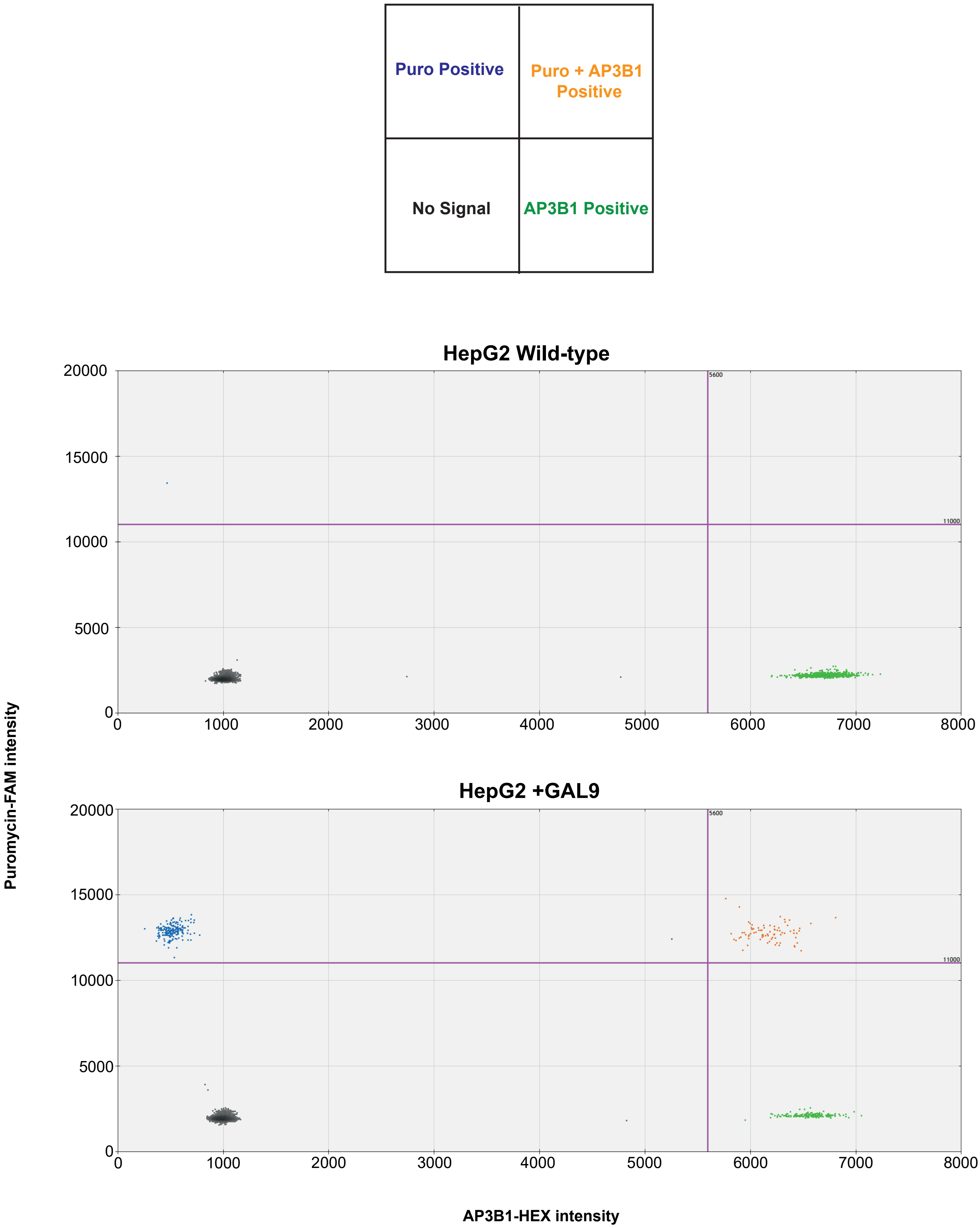

Supplementary Fig. 2 - Droplet digital detection of reporter insertion

Representative droplet digital PCR dot plots for the amplification of puromycin cassette in the HepG2 cell pool, intensity of Puromycin-FAM plotted relative to AP3B1-HEX intensity. Each dot represents a PCR droplet analysed. Pink lines represent the fluorescence intensity thresholds set for data analysis for each fluorophore across samples.

Supplementary Figure 3

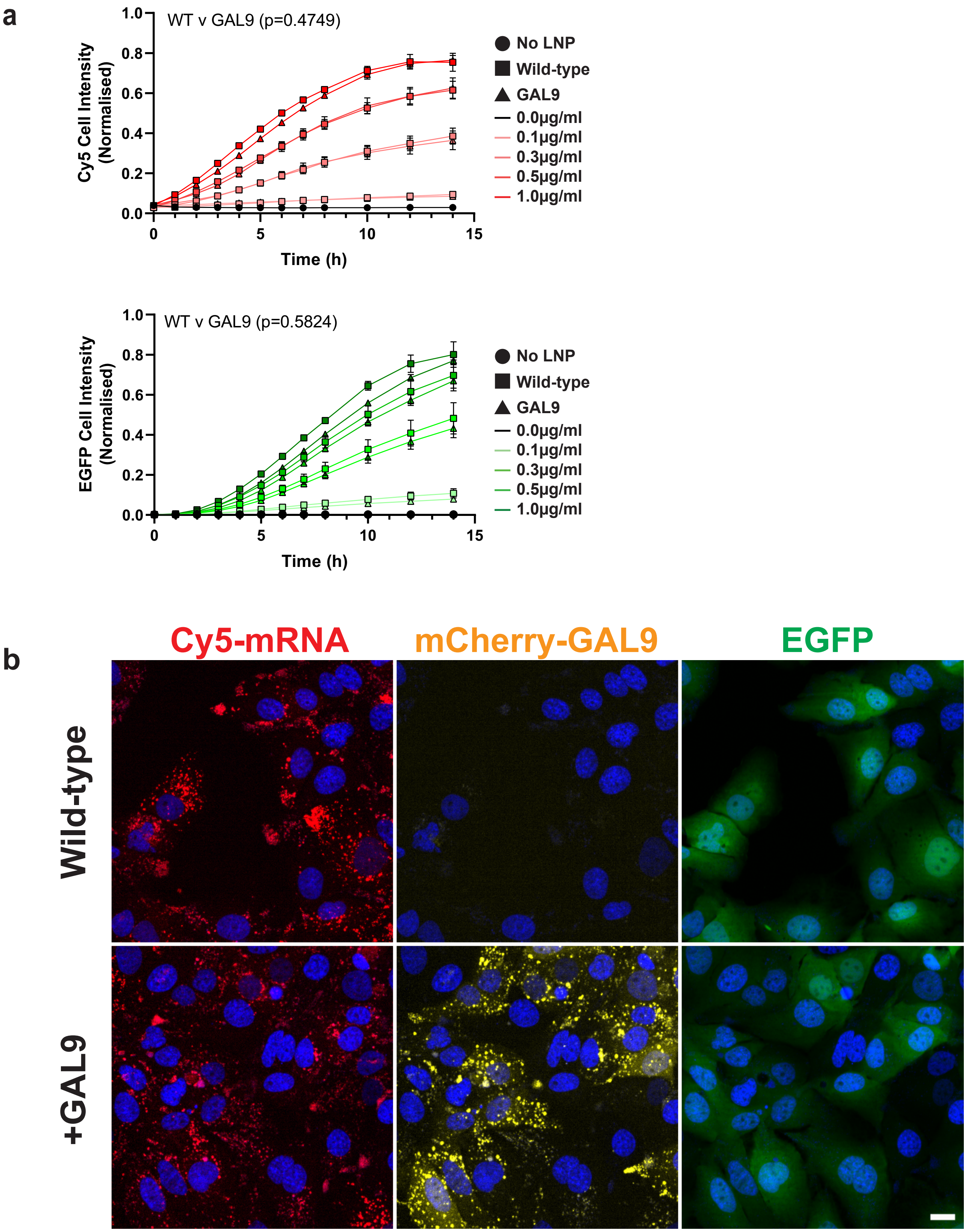

Supplementary Fig. 3 – mCherry-GAL9 expression does not significantly alter MC3-LNP response

a Huh7 wild-type cells or those containing mCherry-GAL9 were dosed with MC3 LNPs from 0-1 µg/ml and imaged by live-cell microscopy. The cellular Cy5 and EGFP intensities were plotted over time and represent normalised means from n=3 independent experiments ± SEM. Significance was determined by two-way ANOVA followed by Sidak's post-test to compare cell lines. **b** Representative live cell images of cells treated as in **a** at 14 h post-LNP dosing, scale bar = 20 µm.

Supplementary Figure 4

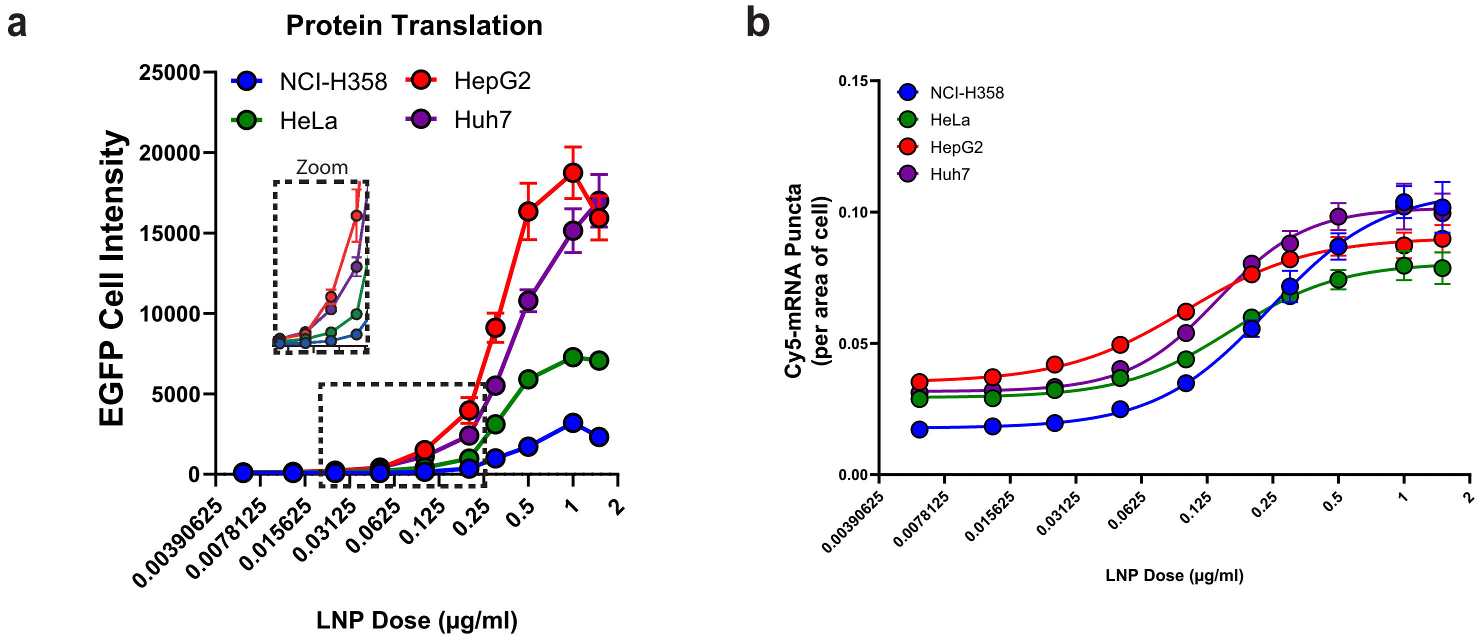

Supplementary Fig. 4 – MC3-LNP dose v Uptake and protein production

a Sum of Cy5 puncta across time were derived (from 0 14 h) and plotted against the MC3-LNP dose. Values represent mean ± SEM for n= 3 independent experiments. Four-parameter logistic curve with variable slope. **b** Plot of mean cellular EGFP intensity ± SEM for different cell lines after 14 h incubation with indicated dose of MC3-LNP from n=3 independent experiments.

Supplementary Figure 5

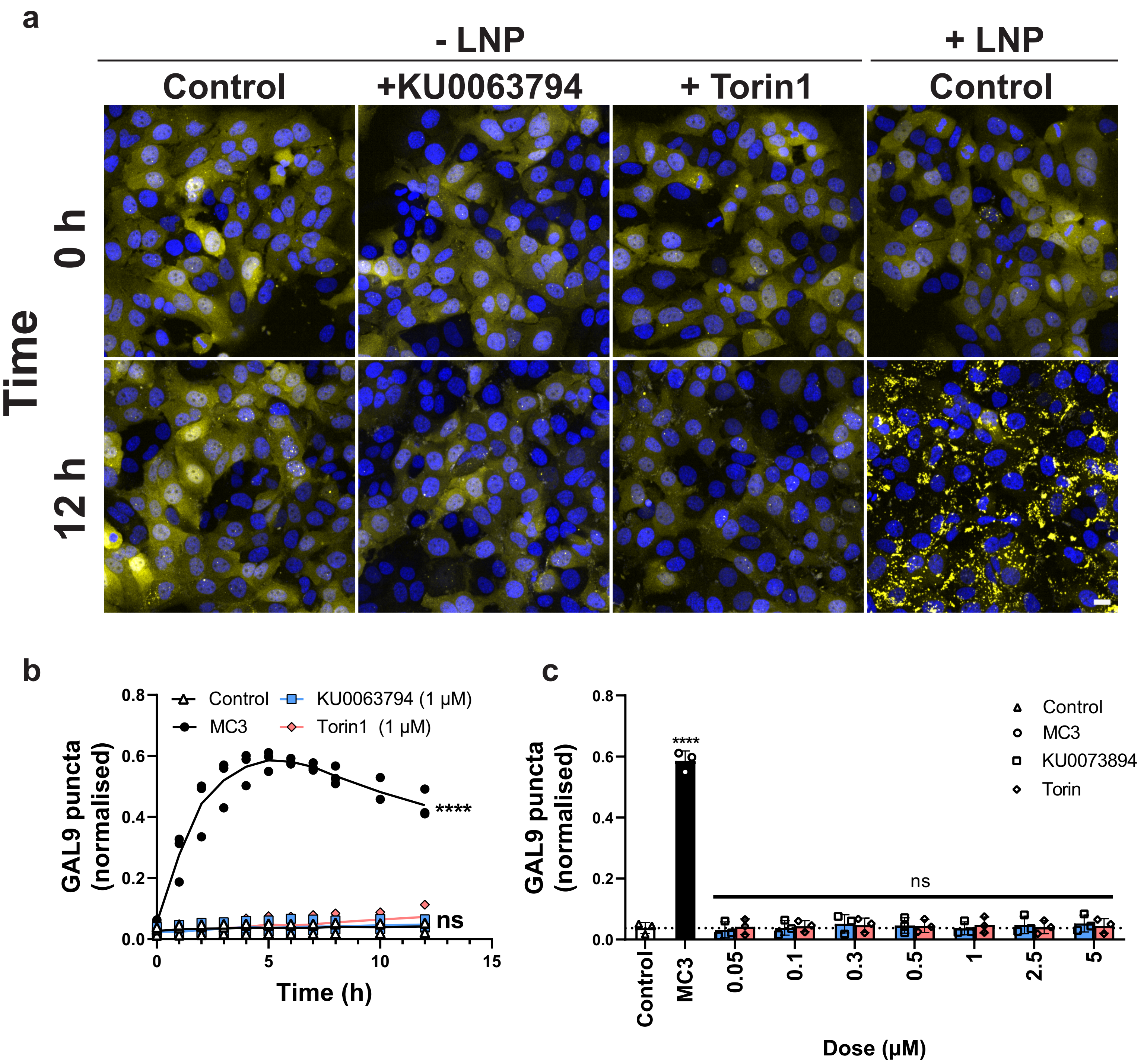

Supplementary Fig. 5 – Autophagy does not significantly induce mCherry-GAL9 objects

a Huh7-GAL9 cells were imaged live in the presence of KU0063794 or Torin1 (both 1 μM) or dosed with MC3-LNP (0.5 μg/ml) for up to 12 h in the presence of 0.5 μg/ml Hoechst 33342. Scale bar = 20 μm. **b** Quantitation of cell mCherry-GAL9 puncta following treatment as in **a** for 0-12 h. Lines represents mean values from n=3 independent experiments **c** Dose range of mTOR inhibitors (0.05 μM – 5 μM) at 5 h post-dosing. Values represent normalised means ± SEM from n=3 independent experiments. Significance was determined in **b**,**c** by two-way ANOVA followed by Dunnett's post-test comparison to the untreated control where ****=p<0.0001 and ns=not significant.

Supplementary Figure 6

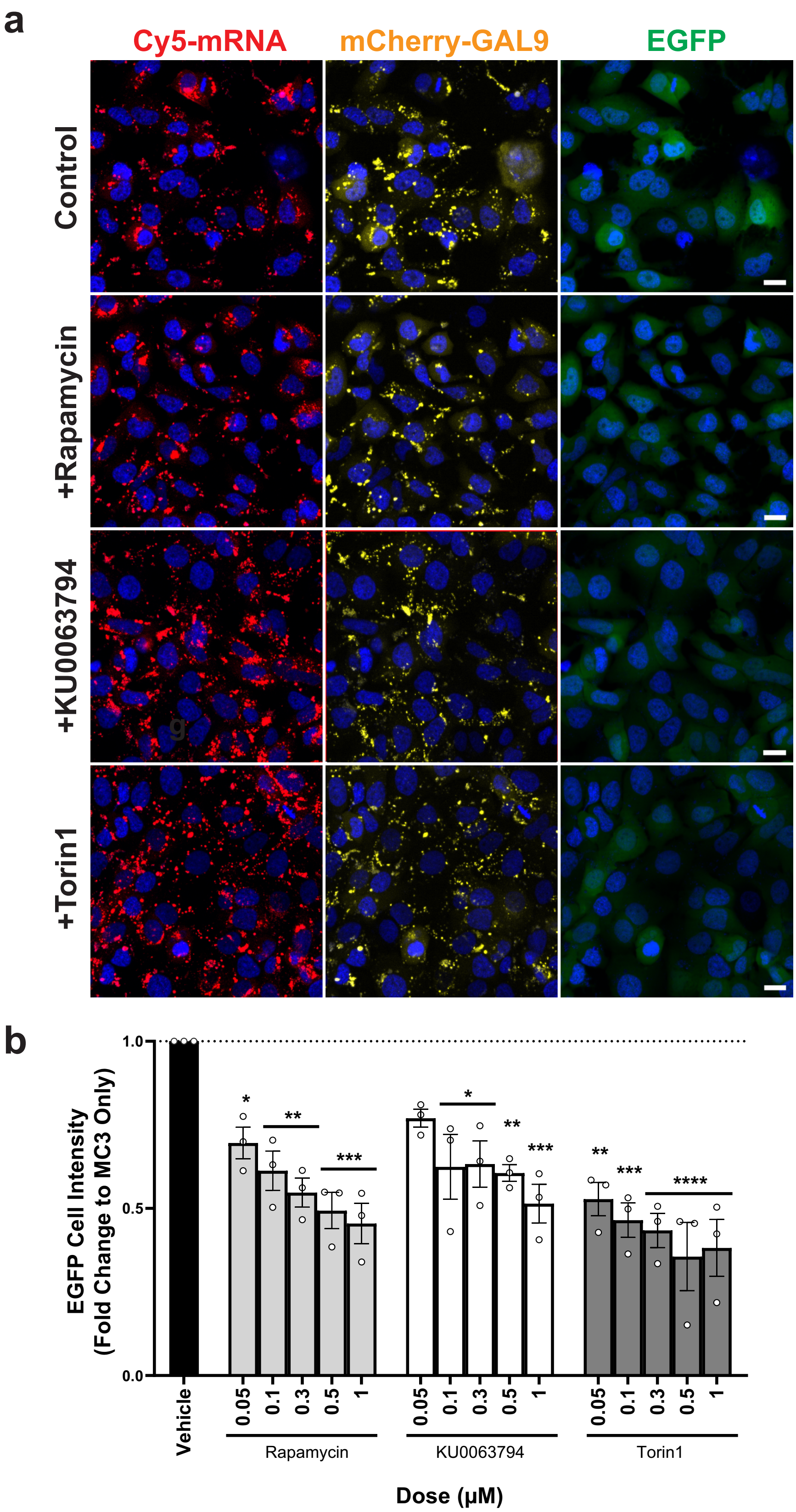

Supplementary Fig. 6 – mTOR inhibitors reduce LNP cargo protein translation

a Representative fluorescence images of Huh7 mCherry-GAL9 cells dosed with 0.5 μg/ml MC3 LNPs and vehicle, Rapamycin (0.1 μM) or KU0063794 (0.1 μM) for 12h. Scale bar = 20 μm. **b** Quantitation of dose range of mTOR inhibitors (0.05μM - 1μM) when treated as in **a**. Values represent normalised means ± SEM from n=3 independent experiments. Significance was determined in **b** by two way ANOVA followed by Dunnett's post-test comparison to the MC3 untreated control where * = p<0.05, **=p<0.01 ***= p<0.001, ****=p<0.0001.

Supplementary Figure 7

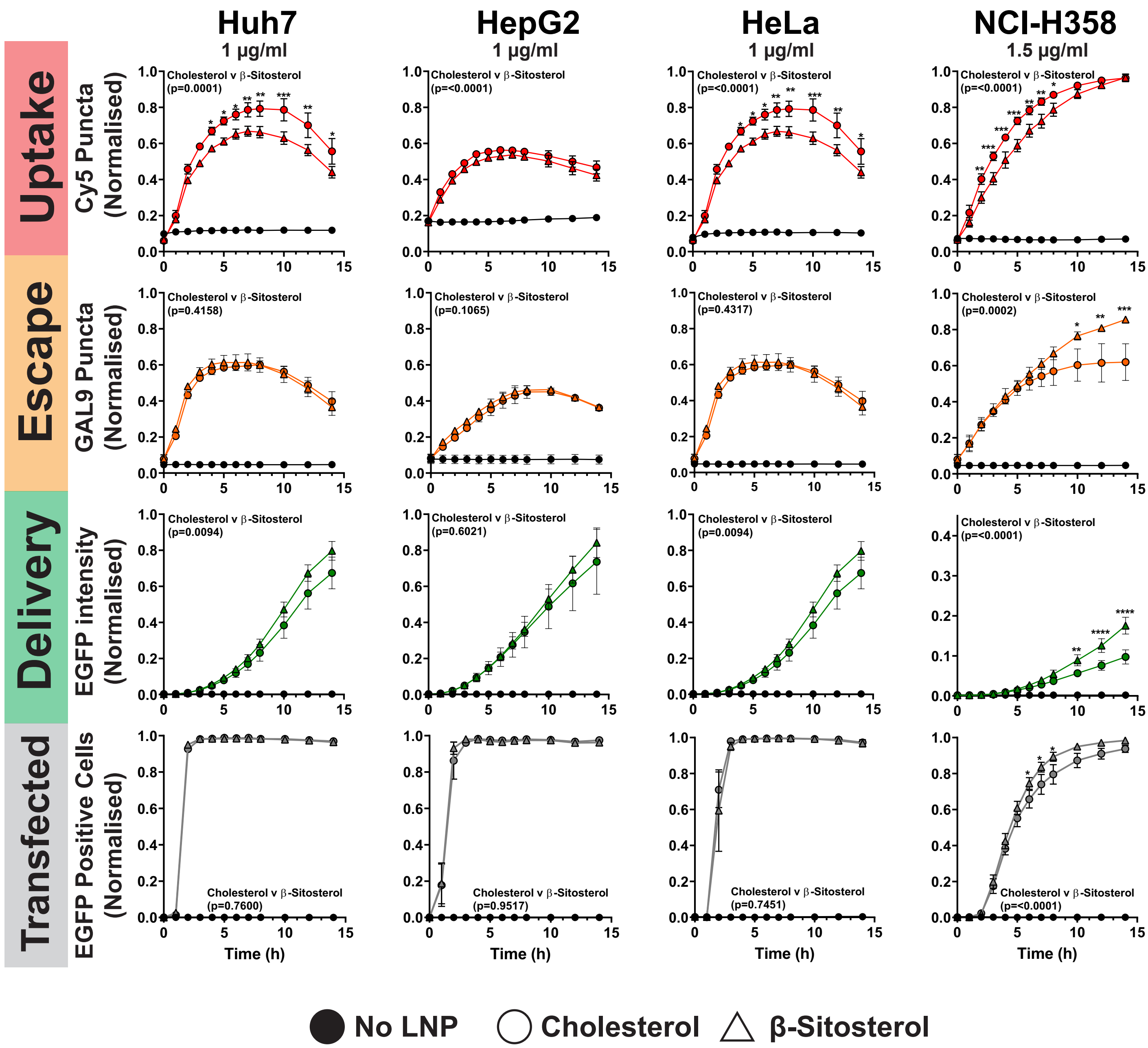

Supplementary Fig. 7 – High doses of β-sitosterol vs cholesterol LNPs

Full time course graphs (0-14 h) of Cy5 puncta, mCherry-GAL9 puncta and EGFP intensity from reporter cell lines dosed with particles at 1 µg/ml (Huh7, HepG2, HeLa) or 1.5 µg/ml (NCI-H358). Values represent normalised means ± SEM from n = 3 independent experiments. Significance was determined by two-way ANOVA followed by Tukey's multiple comparison test where * = p<0.05, **=p<0.01 ***= p<0.001 and ****=p<0.0001.

Supplementary Figure 8

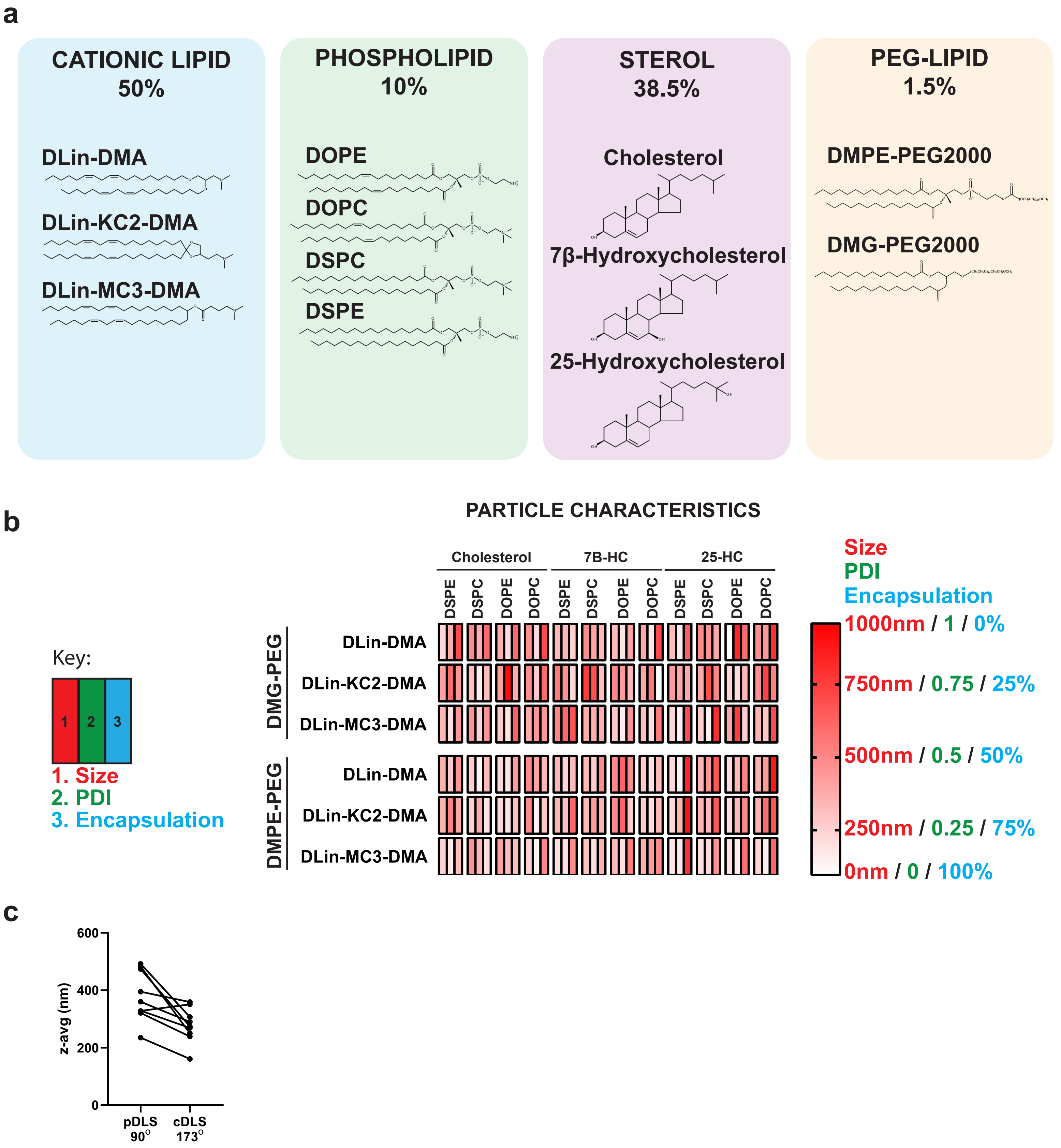

Supplementary Fig. 8 – Characterisation of LNP particle variants

a Overview and comparison of different lipid structures utilised for particle variation in Fig. 6. **b** Heatmap summary of particle characterisation data across z-avg size (1), polydispersity index (2. PDI) and encapsulation % (3) from n=4 independent experiments. **c** comparison of z-avg particle size obtained using a plate-based DLS (pDLS) at 90° reading angle compared to a cuvette DLS (cDLS) at 173° reading angle.

Supplementary Figure 9

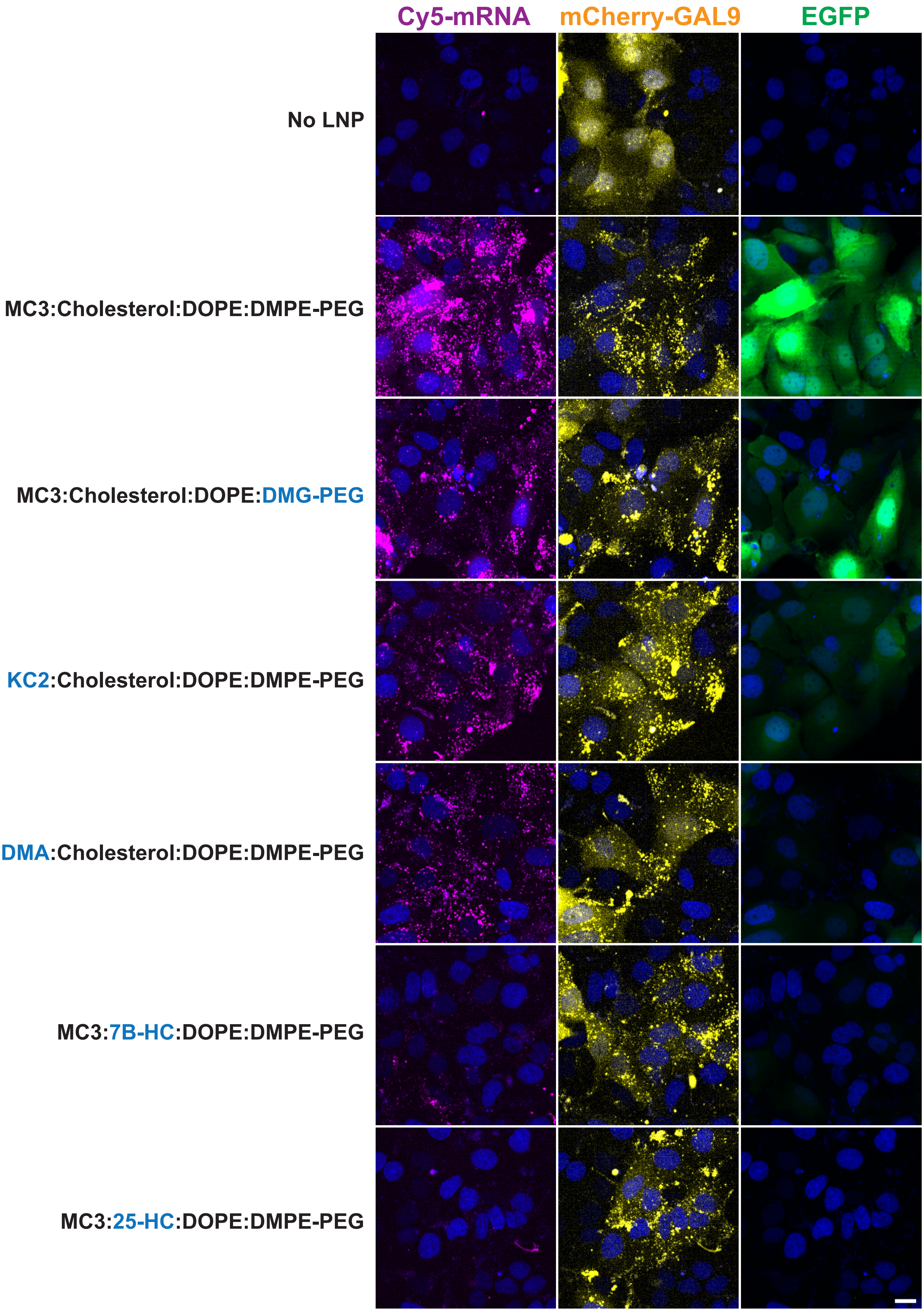

Supplementary Fig. 9 – Screening of LNP particle variants

Representative images from mCherry GAL9 Huh7 cells at 14 h post-dosing of particles modified for Cationic lipid, Sterol or PEG Lipid. Changed component compared to MC3:Cholesterol:DOPE:DMPE-PEG particle is highlighted in blue. Scale bar = 20 µm.

Supplementary Table 1

Cell Line:	Huh7	HepG2	HeLa	NCI-H358
GAL9 v Dose (Fig. 3e)				
Bottom	0.0191	0.04096	0.01407	0.01341
Slope	1.502	2.673	1.643	1.711
Top	0.07763	0.0727	0.05354	0.0484
EC50 (µg/ml)	0.09877	0.2329	0.3722	0.3496
Span	0.05853	0.03174	0.03947	0.03499
R^2	0.9993	0.9917	0.9947	0.9941
Cy5 v GAL9 (Fig. 3f)				
R^2	0.9188	0.573	0.6278	0.8161
Cy5 v Dose (Supplementary Fig. 4b)				
Bottom	0.03167	0.03509	0.02934	0.01771
Slope	2.13	1.54	1.755	1.669
Top	0.1016	0.09031	0.08069	0.1083
EC50 (µg/ml)	0.1397	0.1003	0.1623	0.2408
Span	0.06996	0.05521	0.05135	0.09061
R^2	0.8831	0.8933	0.8842	0.9001

Supplementary Table 1 – Curve/Line Statistics

Supplementary Table 2

Particle Name	Used (Figures)	Composition	Amine:Phosphate (N:P)	Diameter (z-avg, nm)	PDI	Encapsulation	Method
MC3	Fig. 3<br>Fig. S3<br>Fig. S5<br>Fig. S6	50% DLin-MC3-DMA<br>37.5% Cholesterol<br>10% DSPC<br>1.5% DMPE-PEG	~3:1<br>(10:1 w:w)	1 – 82.5<br>2 – 85.2<br>3 – 84.2<br>Avg = 83.9 ± 1.4	0.02<br>0.02<br>0.06<br>Avg = 0.035 ± 0.02	97.7<br>97.0<br>92.6<br>Avg = 95.6% ± 3.1	NanoAssemblr
EB10250	Fig. 4	100% Polymer	8:1	511	0.286	70.6%	Automated Pipetting
EB10970	Fig. 4	100% Polymer	8:1	606	0.104	67.9%	
EB17610	Fig. 4	100% Polymer	8:1	697	0.150	67.6%	
PEG-EB9187	Fig. 4	100% Polymer	8:1	48	0.199	21.0%	
PEG-EB12350	Fig. 4	100% Polymer	8:1	57	0.015	25.3%	
PEG-EB18280	Fig. 4	100% Polymer	8:1	290	0.036	36.2%	
MC3<br>Cholesterol	Fig. 5<br>Fig. S7	50% DLin-MC3-DMA<br>37.5% Cholesterol<br>10% DSPC<br>1.5% DMPE-PEG	~6:1<br>(20:1 w:w)	61.5	0.040	98.1%	NanoAssemblr
MC3<br>β-Sitosterol	Fig. 5<br>Fig. S7	50% DLin-MC3-DMA<br>37.5% β-Sitosterol<br>10% DSPC<br>1.5% DMPE-PEG	~6:1<br>(20:1 w:w)	90.6	0.044	98.4%	NanoAssemblr
Robotic LNP Formulations	Fig. 6<br>Fig. S8<br>Fig. S9	50% Cationic Lipid<br>37.5% Sterol<br>10% Phospholipid<br>1.5% PEG-Lipid	~6:1<br>(20:1 w:w)	See Fig. S8c	See Fig. S8c	See Fig. S8c	Automated Pipetting

Supplementary Table 2 – Particle Parameters
