# Supplementary material for: A high-throughput Galectin-9 imaging assay for quantifying nanoparticle uptake, endosomal escape and functional RNA delivery
Source: Commun Biol. 2021 Feb 16;4:211. doi: 10.1038/s42003-021-01728-8 (PMC7887203; doi:10.1038/s42003-021-01728-8)
Supplement: Supplementary file 3 — Description of Additional Supplementary Files [file 42003_2021_1728_MOESM3_ESM.pdf]

Description of Additional Supplementary Files

File Name: Supplementary Data 1

Description: Within this source data file, we have deposited all of the underlying data used to generate graphs portrayed in Figure 1-6 and Supplementary Figure 3-8. We have also included original western blot and agarose gel images and indicated cropped regions for images displayed in Fig. 1.
